# Sorption capacity of seaweed-like sodium titanate mats for Co^2+^ removal†

**DOI:** 10.1039/d0ra06662a

**Published:** 2020-11-11

**Authors:** Yoshifumi Kondo, Tomoyo Goto, Tohru Sekino

**Affiliations:** The Institute of Scientific and Industrial Research (ISIR), Osaka University 8-1 Mihogaoka, Ibaraki Osaka 567-0047 Japan goto@sanken.osaka-u.ac.jp sekino@sanken.osaka-u.ac.jp +81-6-6879-8439 +81-6-6879-8436; Division of Materials and Manufacturing Science, Graduate School of Engineering, Osaka University 2-1 Yamadaoka, Suita Osaka 565-0871 Japan

## Abstract

The development of new technologies for securing and recycling water resources are in high demand. A key focus of these technologies is the development of various ion exchangers or adsorbents that are used for the purification of aqueous solutions. Layered sodium titanate is one of the cation exchangers utilised in the removal of heavy metals and radionuclides from wastewater. To enhance the removal efficiency, the precise design of the crystal morphology, structure, and chemical composition is important. Herein, we synthesised a unique seaweed-like sodium titanate mat (SST) using a template-free alkaline hydrothermal process. The Co^2+^ sorption capacity of SST was investigated by batch testing with cobalt(ii) nitrate. SST, which was synthesised from titanium sulphate in a 10 M NaOH solution at 200 °C, had a seaweed-like structure composed of randomly distributed nanofibres of layered sodium titanate that is approximately 9 nm in diameter. The crystal shape changed from roundish crystals to fibrous crystals as the hydrothermal reaction period increased. The Co^2+^ sorption isotherm of SST was fitted with the Langmuir isotherm model and the maximum sorption density was 1.85 mmol g^−1^. The selectivity of the Co^2+^ sorption on SST was high in comparison to that of Ca^2+^ and Mg^2+^. Herein, the Co^2+^ sorption mechanisms of SST were studied in comparison with commercially available sodium titanate. Results show that controlling the crystal morphology, structure, and Na concentration of the layered titanate that can be ion-exchanged determines the cation sorption properties of sodium titanate.

## Abstract 2

The seaweed-like sodium titanate mat has a high Co^2+^ sorption capacity *via* the ion-exchange reaction due to its crystal structure, low crystallinity, and morphology.

## Graphical abstract

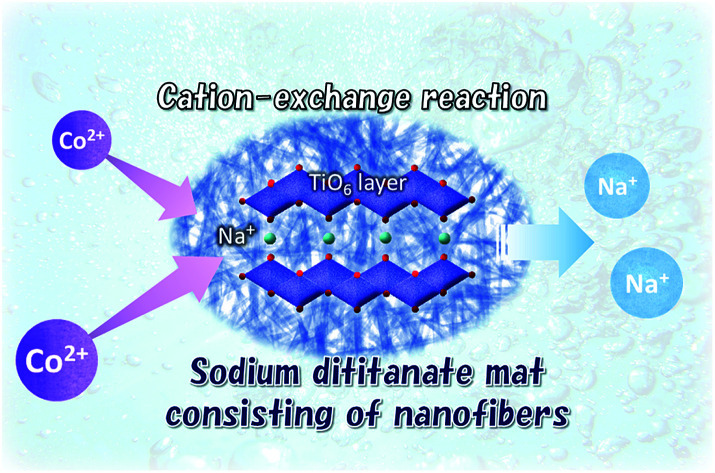

## Introduction

For the conservation of water resources, the development of water management and water treatment technologies are important. Heavy metals, radionuclides, harmful anions, and volatile organic compounds are water pollutants released from households, industrial, and nuclear facilities. For instance, cobalt(ii) ions (Co^2+^) are one of the toxic metals, a radioisotope that affect our health and the environment. In general, the cobalt concentration in drinking water is less than 1–2 ppb.^1^ Cobalt ions are one of the essential elements used by the human body for metabolism. However, several health hazards result from high concentrations of cobalt found in wastewaters discharged from nuclear power plants and various industrial activities. Although the utilization, storage, and disposal of Co ions are strictly controlled, there is a need to develop a technology that removes Co ions from the environment in order to prevent health hazards and environmental pollution. This has led to the proposal of many purification techniques.^2,3^ In recent years, some organic or inorganic materials have been proposed as adsorbents for Co^2+^ removal from wastewater.^4–13^ Furthermore, several heavy metals and radionuclides can be removed using inorganic ion exchangers.^14^ Sodium titanate compounds are used as a purification material for the removal of heavy metal ions and radionuclides from industrial wastewater. For example, hydrous sodium titanate is used for the removal of radioactive Sr^2+^ from the wastewater of the Fukushima Daiichi Nuclear Power Station.^15,16^ Sodium titanate, which is a two-dimensional layered structure, has various chemical compositions and crystal structures.^16–22^ These structures enable sodium titanate to recover and immobilise cations of radionuclides and heavy metals by ion-exchange with cations (Na^+^) in the interlayer of layered titanates. Previously, the ion-exchange properties of layered titanates were investigated using various mono-, bi-, and tri-valent cations, such as Cs^+^, Sr^2+^, Cu^2+^, Pb^2+^, and Eu^3+^.^15,16,20–22^

The crystallographic properties of the sorbent are directly related to sorption mechanisms, the design of the surface area, crystal structure, and chemical composition of the sorbents. These are key factors in the enhancement of the sorption efficiency of the target cations. Therefore, unique morphological effects on the ion-exchange and adsorption properties of sodium titanate have been investigated to determine the optimal material design and material process design.^21^ Many methods have been proposed for the synthesis of layered titanates with various morphologies. Among them, hydrothermal synthesis is one of the effective synthetic methods. Many of these methods also use an alkaline treatment of the precursor materials.^17,18,21,23,24^ In the hydrothermal synthesis process, it is known that the characteristics of the product are affected by the hydrothermal conditions and properties of the starting materials, such as titanium propoxides^17^ and various TiO_2_.^18,21,23,24^

Although the long fibre and needle-shaped crystals of sodium titanates are easily formed with alkaline hydrothermal synthesis, these crystals with micro-order have low specific surface areas and high crystallinity. The morphology, such as a large-sized long fibre, causes a reduction in the sorption density of the ion-exchange reaction. The material design, which like a non-woven fabric composed of nanofibres, that achieves high permeability and high sorption properties is required for ion exchangers for water purification.

In the present study, a seaweed-like sodium titanate (SST) mat with a unique morphology was synthesised using titanium sulphate solution by a simple alkaline hydrothermal synthesis method without the addition of surfactants or templates. The crystal phase, chemical composition, and morphology of SST were compared with commercially available sodium trititanate (Na_2_Ti_3_O_7_) by a physicochemical method, and the formation behaviour of SST was investigated by examining the crystal structure for each heating time. The sorption capacity and behaviour of Co^2+^ were investigated by batch testing using a 0.2–4.0 mM cobalt(ii) nitrate solution, and the state of Co^2+^ in the titanate structure was investigated by X-ray analysis. In addition, the selectivity of Co^2+^ sorption on SST was investigated using a test solution of Co^2+^, Ca^2+^, Mg^2+^, and Na^+^.

## Experimental

### Hydrothermal synthesis of seaweed-like sodium titanate

A titanium sulphate (Ti(SO_4_)_2_, FUJIFILM Wako Pure Chemical Corporation, Osaka, Japan) solution was used as the starting material. A 10 M sodium hydroxide (NaOH, FUJIFILM Wako Pure Chemical Corporation, Osaka, Japan) solution was prepared. 6.87 mL of 30wt% Ti(SO_4_)_2_ solution and 9.13 mL of ultra-pure water were added to 40 mL of 10 M NaOH. The mixed solution was transferred to a 100 mL Teflon liner, which was then sealed and placed in an autoclave for 48 hours at 200 °C. The product was filtered, washed three times with ultra-pure water, and freeze-dried to obtain a powder sample. Furthermore, to investigate the formation of the seaweed-like structure, SST samples were synthesised at various hydrothermal heating times (0 (before hydrothermal synthesis), 1, 2, 3, 6, 12, and 24 h) using the same raw material composition. Powder samples were collected by the same procedure, as described above. They were marked as SST_*t*, where *t* is the heating time.

### Co^2+^ sorption test

The Co^2+^ sorption test was performed using a 0.2–4.0 mM cobalt nitrate solution. Cobalt(ii) nitrate hexahydrate (Co(NO_3_)_2_·6H_2_O, FUJIFILM Wako Pure Chemical Corporation, Osaka, Japan) was dissolved in ultra-pure water and diluted to the various concentrations of test solutions without pH adjustment. Commercially available sodium metatitanate (Na_2_Ti_3_O_7_, Sigma-Aldrich Japan, Tokyo, Japan) was used as a control sample for the sorption test. A 0.01 g sample of the sorbent, either synthesised seaweed-like sodium titanate or sodium metatitanate, was transferred to a centrifuge tube and 20 mL of 0.2–4.0 mM cobalt nitrate test solution was added. The tube was shaken at 150 rpm in a mechanical shaker (BioShaker BR-43FL MR, TAITEC CORPORATION, Saitama, Japan) at 25 °C for one day. Subsequently, the sorbents and solution were separated by filtration, and the residues were analysed by physicochemical methods after freeze-drying (Freeze Dryer, FDU-2200, TOKYO RIKAKIKAI CO., LTD., Tokyo, Japan). In addition, the selectivity test of ion sorption was performed using Co^2+^, Ca^2+^, Mg^2+^, and Na^+^. The mixed solution was adjusted to comprise a concentration of 1 mM for each of the above-mentioned ions, and was prepared using Co(NO_3_)_2_·6H_2_O, calcium nitrate tetrahydrate (Ca(NO_3_)_2_·4H_2_O, FUJIFILM Wako Pure Chemical Corporation, Osaka, Japan), magnesium chloride hexahydrate (MgCl_2_·6H_2_O, FUJIFILM Wako Pure Chemical Corporation, Osaka, Japan), and sodium chloride (NACALAI TESQUE, INC., Kyoto, Japan). The sorption test was conducted by using the procedure described above.

### Characterisation

The ion concentrations of Co^2+^ Ca^2+^, Mg^2+^, and Na^+^ remaining in the solution after the sorption test were measured using inductively coupled plasma optical emission spectrometry (ICP-OES; Optima 8300, PerkinElmer Japan Co., Ltd., Yokohama, Japan). A wavelength-dispersive X-ray fluorescence spectrometer (XRF, ZSX-100e, Rigaku Corporation, Tokyo, Japan) in EZ scan mode was also used to investigate the concentration of the initial Na in the samples, and the Co concentration in the residue after the test. The pH of the solution before and after sorption testing was measured using a pH meter (D-52, HORIBA, Ltd., Kyoto, Japan). The crystal size, morphology, and microstructure of the products were observed using ultra-high resolution scanning electron microscopy (SEM, SU9000, Hitachi High-Technologies Corporation, Tokyo, Japan) and transmission electron microscopy (TEM, JEM-2100, JEOL Ltd., Tokyo, Japan). The average thickness of the SST mat was calculated using atomic force microscopy (AFM, VN-8010, KEYENCE Corporation, Osaka, Japan). The crystal phase of the products was identified by powder X-ray diffraction (XRD, D8 ADVANCE, Bruker AXS, Germany) using Cu-Kα radiation at 40 kV and 40 mA. The adsorption–desorption isotherms of N_2_ gas were measured at 77 K using a Surface Area & Pore Size Analyser (NOVA 4200e, Quantachrome Instruments Japan G. K., Kawasaki, Japan) after each sample was evacuated at 378 K for 3 h. From the isotherm, the specific surface area of the products was calculated using the multi-point Brunauer–Emmett–Teller method. The zeta potential of samples was measured using the electrophoretic method on an analyser (Zetasizer Nano ZS, Malvern Instruments Ltd, Malvern, UK). Quick-scanning X-ray absorption fine structure (XAFS) spectra of the Ti K-edge and Co K-edge were obtained using the ionisation chamber in transmission mode on the Kyushu University Beamline (BL06) of the Kyushu Synchrotron Light Research Center (SAGA-LS; Tosu, Japan). The Ti K-edge extended XAFS (EXAFS) spectra were collected over a photon energy range of 4.6 to 6.2 keV. For the Co K-edge, the EXAFS spectra were measured in the range of 7.6 to 7.9 keV. The spectra obtained were analysed using REX2000 software (Rigaku Co., Tokyo, Japan). Anatase (TiO_2_, FUJIFILM Wako Pure Chemical Corporation, Osaka, Japan), Rutile (TiO_2_, FUJIFILM Wako Pure Chemical Corporation, Osaka, Japan), and sodium metatitanate were used as reference samples for the Ti K-edge. Additionally, cobalt(II, III) oxide (Co_3_O_4_, NACALAI TESQUE, INC., Kyoto, Japan), Co(NO_3_)_2_·6H_2_O, and cobalt hydroxide (Co(OH)_2_, FUJIFILM Wako Pure Chemical Corporation, Osaka, Japan) were used as reference samples for the Co K-edge.

### Theoretical analysis of the sorption isotherm of Co^2+^

The results of the sorption test were analysed using the following method. The removal efficiency (%) of Co^2+^ from the test solution was calculated as follows:1
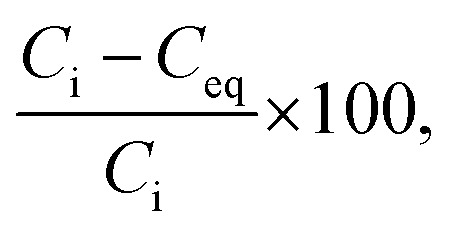
where *C*_i_ is the initial concentration of Co^2+^ (mmol L^−1^) and *C*_eq_ is the equilibrium concentration of Co^2+^ in the test solution (mmol L^−1^). In addition, the sorption isotherm of Co^2+^ on the sorbents was analysed using the Langmuir equation. *C*_i_ and *C*_eq_ are related by the following expression:2
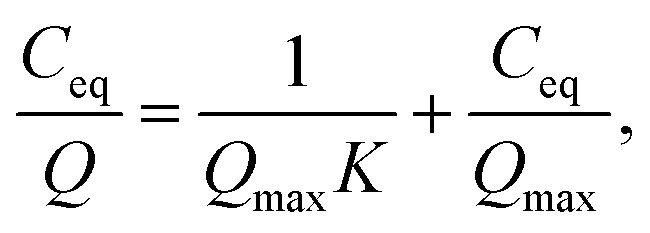
where *Q* is the amount of Co^2+^ absorbed per gram (mmol g^−1^), *Q*_max_ is the maximum amount of Co^2+^ adsorbed on the sorbent (mmol g^−1^), and *K* is a constant related to the adsorption rate coefficient.

The theoretical ion-exchange capacity per gram (IEC, mmol g^−1^) was calculated as follows:3
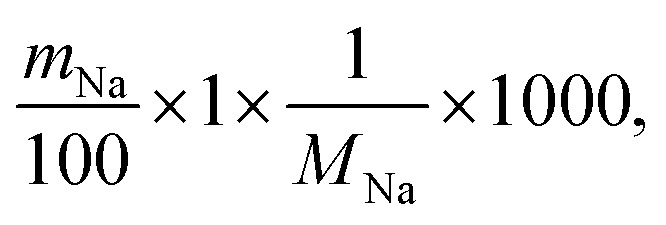
where *m*_Na_ (wt%) is the Na concentration of samples obtained by ICP-OES analysis, and *M*_Na_ is the atomic weight of Na (g mol^−1^).

## Results and discussion

### Characterisation of the SST mat

**Fig. 1:**
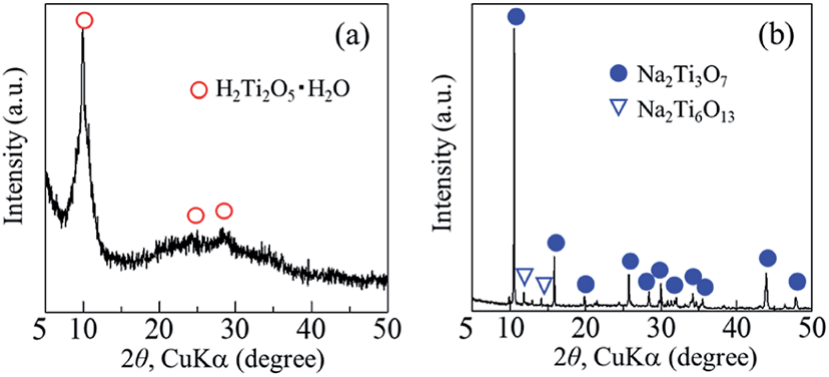
Powder XRD patterns of (a) synthesised products and (b) commercially available sodium metatitanate.

**Fig. 2:**
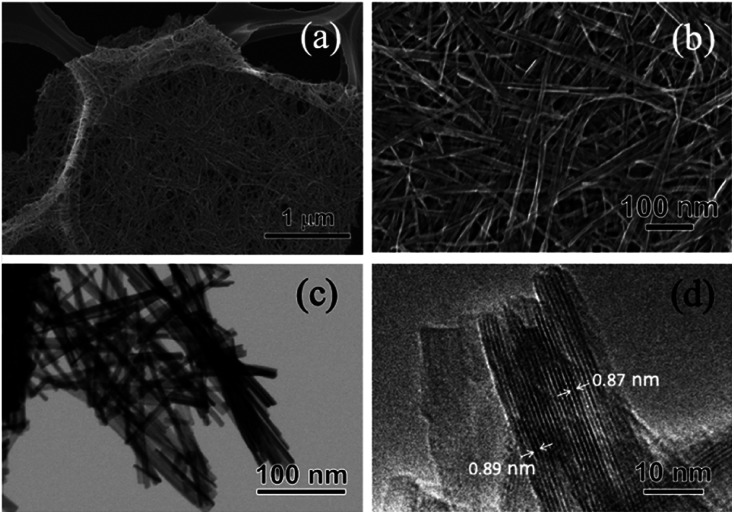
(a and b) SEM, (c) STEM, and (d) TEM images of the SST mat synthesised by hydrothermal treatment, maintaining a temperature of 200 °C for 48 h.

The crystal phase, structure, elemental composition, and morphology of the synthesised product after maintaining a temperature of 200 °C for 48 h were investigated using various physicochemical methods. Fig. 1a shows the powder XRD pattern of the product synthesised by the one-step hydrothermal treatment. All of the peaks of the product were assigned to a layered dititanate phase (H_2_Ti_2_O_5_·H_2_O) (PDF no. 00-047-0124), and the diffractogram had relatively broad peaks. Peaks of byproducts, such as sodium sulphate, were not observed. The *d*-value of 200, which shows the interlayer distance of sodium titanate, was calculated to be 0.89 nm using the maximum peak at 2*θ* = 9.9°. The XRD pattern of commercially available sodium metatitanate (TC) is shown in Fig. 1b. Several high-intensity peaks of Na_2_Ti_3_O_7_ (PDF no. 00-031-1329) and a few low-intensity peaks of Na_2_Ti_6_O_13_ (PDF no. 00-037-0951) were detected (Fig. 1b). The *d*-value of 001, which shows the interlayer distance of Na_2_Ti_3_O_7_, was calculated to be 0.83 nm using the maximum peak at 2*θ* = 10.6°. The *d*-value of the synthesised product was slightly larger than that of TC, as the interlayer distance of dititanate is larger than that of trititanate due to a difference in the crystal structures.^17^ From the SEM images of the synthesised sodium titanate (Fig. 2a and b), a unique mat structure was observed, which was similar to non-woven fabric and composed of nanofibres with a diameter of approximately 8.9 nm. It has been known that the layered sodium titanate has various morphologies, such as nanofibre, plate, and tube structures.^17–25^

A uniform nanofibre structure is shown in the scanning transmission electron microscopy (STEM) image of the synthesised SST (Fig. 2c). Fig. 2d shows the TEM image of a layered structure of nanofibres with a lattice distance of the interlayers of approximately 0.89 nm. These results agree with the interlayer distance calculated using a *d*-value of 200 obtained by XRD analysis. From the AFM observation (Fig. S1†), the average thickness of the SST mat was 129 nm.

**Fig. 3:**
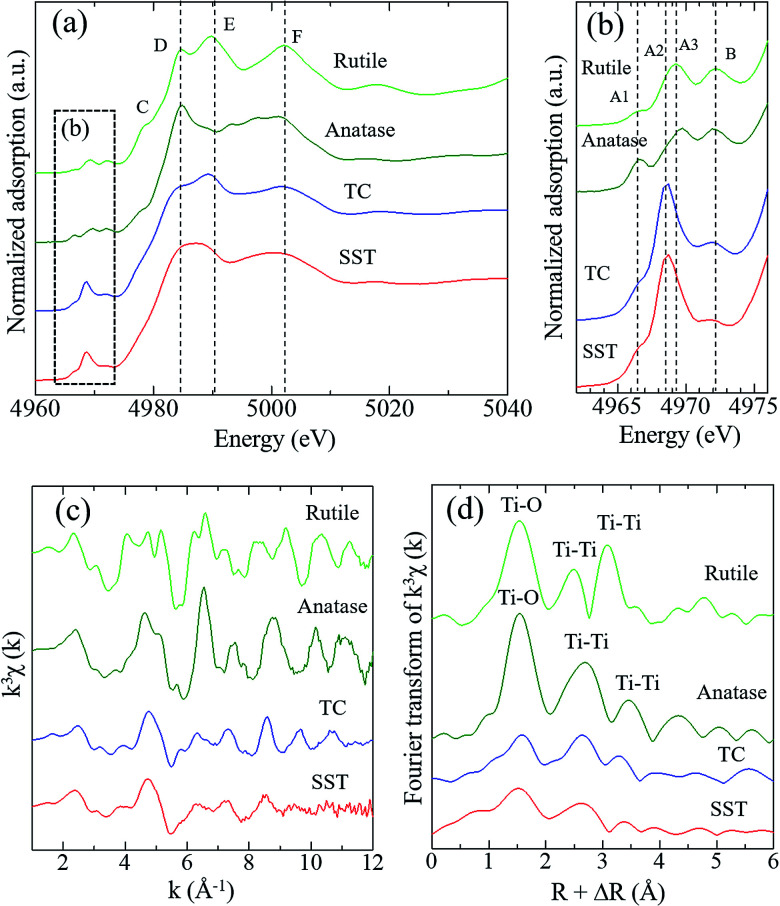
Spectra of (a and b) Ti K-edge XANES of SST, rutile, anatase, and TC, (c) the Ti K-edge *k*^3^-weighted EXAFS and (d) Fourier transform EXAFS of standard reagents and SST. The radial distribution functions were not corrected for phase shift.

Fig. 3 shows the Ti K-edge of the X-ray absorption near-edge structure (XANES) spectra of titanium compounds. The XANES spectrum of the synthesised product, SST, is more similar to TC than rutile or anatase. The white line peaks of D, E, and F, which are assigned to the 1s → 4p transition, agree with previous reports of rutile and anatase.^26^ The typical triple pre-edge peaks of A1, A2, A3, and B of anatase and rutile, which are shown in Fig. 3b, are related to the 3d–4p and 4s–4p hybridised states of the Ti atom.^26,27^ The peak intensities of A2 in TC and SST were higher than that of rutile and anatase. Previous studies have shown that the XANES spectra of the layered titanate have an A2 peak with a high intensity, which indicates the presence of the five-order coordination of Ti in the layered titanate structure.^28^ In contrast, the Ti atoms of rutile and anatase are coordinated with six oxygen atoms. Therefore, the Ti atoms of SST and TC are five-order coordinated complexes of Ti. As shown in Fig. 3c, the Ti K-edge EXAFS spectrum of SST is more similar to TC than rutile and anatase. The layered titanates of TC and SST were indicated by the weak amplitude. The FT-EXAFS spectra of all titanates have a peak at 2.4–3.2 Å^−1^, corresponding to the Ti–O first coordination (Fig. 3d). Therefore, the results of the XAFS measurements indicate that the SST sample is layered sodium titanate. In addition, the specific surface area of SST and TC obtained from the N_2_ adsorption–desorption isotherms (Fig. S2a†) were 158.3 and 1.688 m^2^ g^−1^, respectively. The surface area of SST is approximately 100-fold larger than TC. This is because SST has a mat-like morphological structure, whereas TC has a large-sized rod crystal morphological structure (Fig. 2 and S2b†). The zeta potential against the pH value of SST is higher than that of TC in the range of pH = 4–11 (Fig. S3†).

### Formation behaviour of the SST mat

**Fig. 4:**
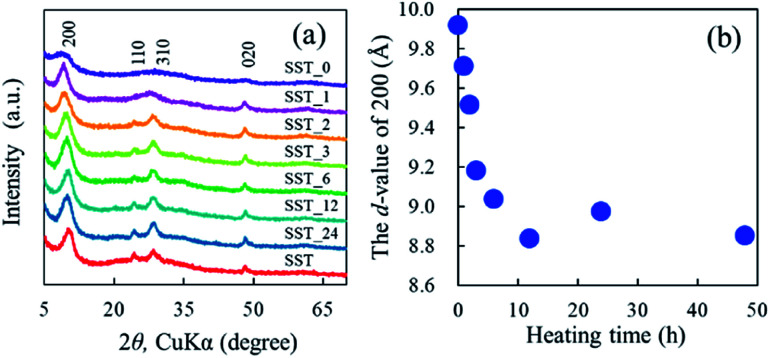
(a) Powder XRD patterns of SST_*t* samples hydrothermally treated at various times, and (b) changes in the Interlayer distance of SST_*t* samples depending on the synthesis time.

The formation behaviour of SST *via* hydrothermal synthesis was investigated by changing the reaction time. This was important because the reaction temperature might affect the crystal phase, morphology, and size of the final product by changing the reaction rate and equilibrium condition. Fig. 4a shows the XRD patterns of the SST_*t* samples. All peaks on the diffractogram were assigned to H_2_Ti_2_O_5_·H_2_O, as observed in the X-ray diffractogram of SST. As the heating time increased, their crystalline also increased until the crystallinity peaked at a heating time of 12 h. The *d*-value of 200 (*d*_200_) of the SST_*t* samples decreased until 12 h (Fig. 4b). Therefore, the crystal structure of SST was formed in at least 12 h.

**Fig. 5:**
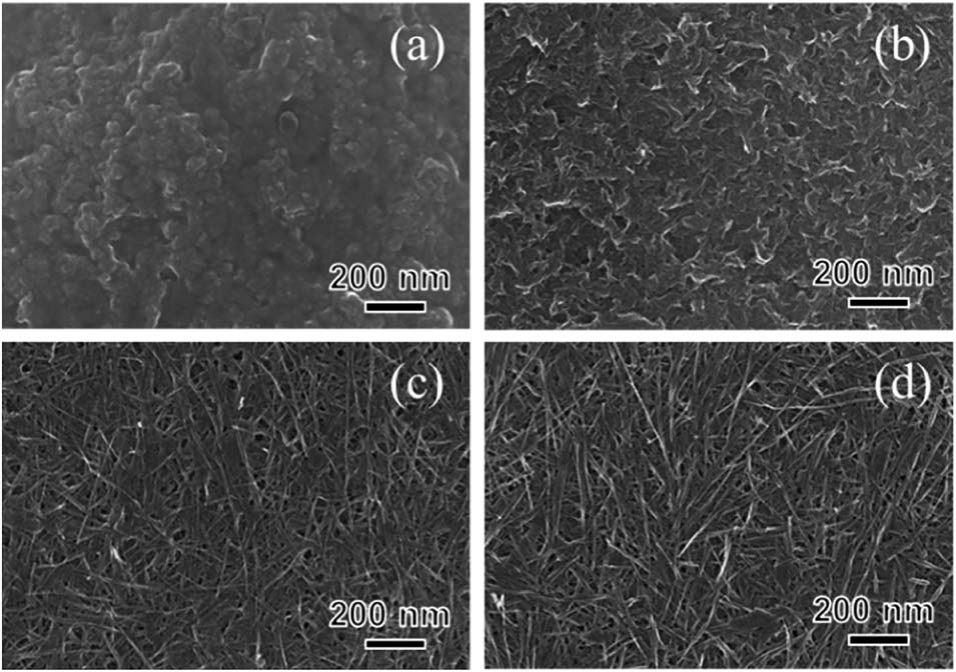
SEM images of (a) SST_0, (b) SST_1, (c) SST_2, and (d) SST_3 samples.

In the case of SST_0, aggregates consisting of nanoparticles were observed, as can be observed in the SEM images of the SST_*t* samples (Fig. 5 and S4†). A mixture of nanoparticle and nanofibre was observed in the SST_1 sample. As the hydrothermal heating time increased, the number of nanoparticles decreased, whereas that of the nanofibres increased (Fig. 5). For samples with a synthesis time above 6 h (SST_12, 24, and 48), nanofibres were only observed, as shown in Fig. S4.† From more macroscopic SEM images, a unique mat structure was formed after hydrothermal synthesis, except for the case of SST_0 (Fig. S4†).

The porous structure and specific surface area (SSA) of the samples were determined using N_2_ adsorption–desorption measurement. All samples had Type II isotherms (Fig. S5†). From increasing in the range of *P*/*P*_0_ > 0.8, the number of the macropores increased as the hydrothermal synthesis time increased until it peaked at a hydrothermal synthesis time of 12 h. The result agreed with the SEM observation (Fig. 5 and S4†) because the macropore structures were made up of overlapping nanofibres, and all nanostructures changed within 12 h. The SSA of the SST_*t* samples is summarised in Table S1.† The SSA dramatically increased at the initial stage of the reaction. After the heating time exceeded 2 h, the SSA remained constant regardless of an increase in the heating time. These results indicate that the changes in the nanostructure had a greater effect on the SSA of the SST_*t* samples compared with the macropore structure.

In addition, the Ti K-edge XANES spectra were acquired to investigate the titanium coordination state of the SST_*t* samples (Fig. S6a and b†). Their pre-edge peaks indicated the presence of five-order coordination of Ti in the SST_*t* samples.^28,29^ The EXAFS spectra (Fig. S6c†) and FT-EXAFS spectra (Fig. S6d†) of all samples were similar to those of sodium titanate. The intensity of the Ti–O first coordination peak increased as the heating time increased (Fig. S6d†). This is because a long-range ordered structure was formed due to the improved crystallinity of sodium titanate with increased heating time as indicated in the XRD patterns (Fig. 4).

**Fig. 6:**
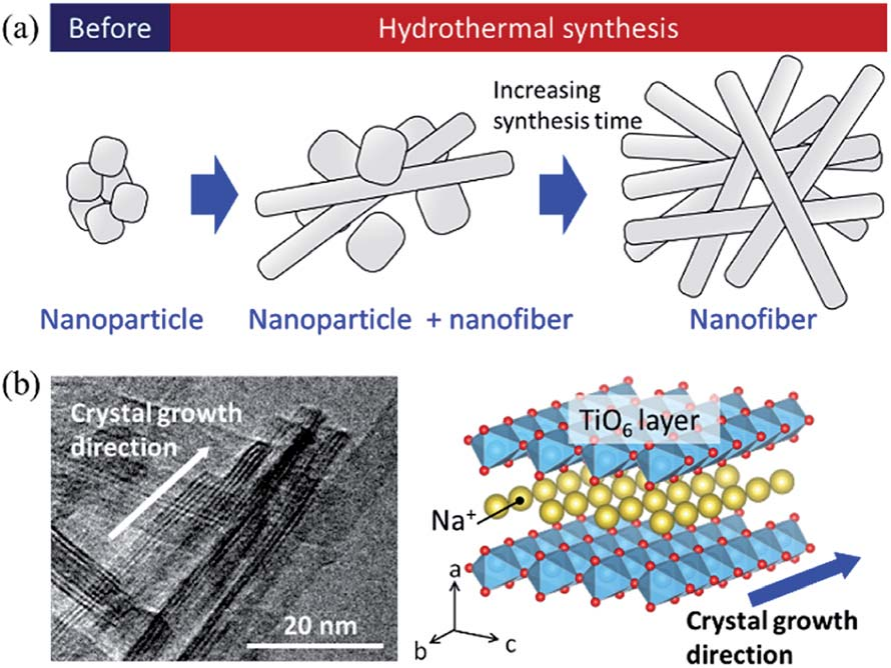
(a) Schematic image of the formation of sodium dititanate nanofibre by hydrothermal synthesis, and (b) comparison of the growth direction between the TEM image and crystal structure model.

Based on the SEM observation and X-ray analysis, a schematic of the formation behaviour of the SST mat is shown in Fig. 6. Before hydrothermal synthesis, amorphous sodium titanate nanoparticles were formed at the time when the solution was mixed (Fig. 6a). Then, non-uniform and roundish-shaped nanocrystals were observed. This suggests that the dissolution–precipitation reaction of the nanocrystals occurred at the initial stage of hydrothermal treatment due to an increase in temperature. Morphological changes to the nanofiber structure and layer reconstruction of the product occurred as the heating time of the hydrothermal treatment increased. These changes result from the exposure of the stable crystal plane, which minimised the interfacial free energy.

Several researchers have reported the relationships between the alkaline condition and crystal growth of the layered titanates on the hydrothermal synthesis.^17,30,31^ Zhao *et al.* (2013) reported that the sodium ion (Na^+^) concentration and solution pH are the important factors that determine the composition of the titanate compounds. In addition, a high Na^+^ concentration is required to maintain the layer structure.^17^ Hence, in this study, a layered sodium titanate was formed at a highly alkaline condition. In addition, the formation of the fibre-, needle-, and plate-shaped crystals of the layered sodium titanates have been reported.^17,30^ Zhang *et al.* (2010) reported that layered sodium titanates easily grow in the [010] direction^31^ because the chemical bonding of the edge- or corner-shared TiO_6_ layer along the [010] and [001] directions is stronger than the stacked layer along the [100] direction. Therefore, due to the difference in the stability of the crystal layer, the crystal growth rate of the [010] direction is considered to be faster than that of the [100] direction. The TEM image of SST is in agreement with this claim (Fig. 6b). In addition, a unique seaweed-like mat comprising nanofibres might be formed *via* a freeze-drying process. This is because nanofibres are two-dimensionally aggregated when subjected to freezing along with rotation. These structural changes upon freezing were confirmed by preliminary examinations. Although a detailed examination is necessary, it can be inferred that this mat structure can be formed by chemical interaction. This is because the structure cannot be formed by micro-sized fibres.

### Co^2+^ sorption capacity of SST and TC

**Fig. 7:**
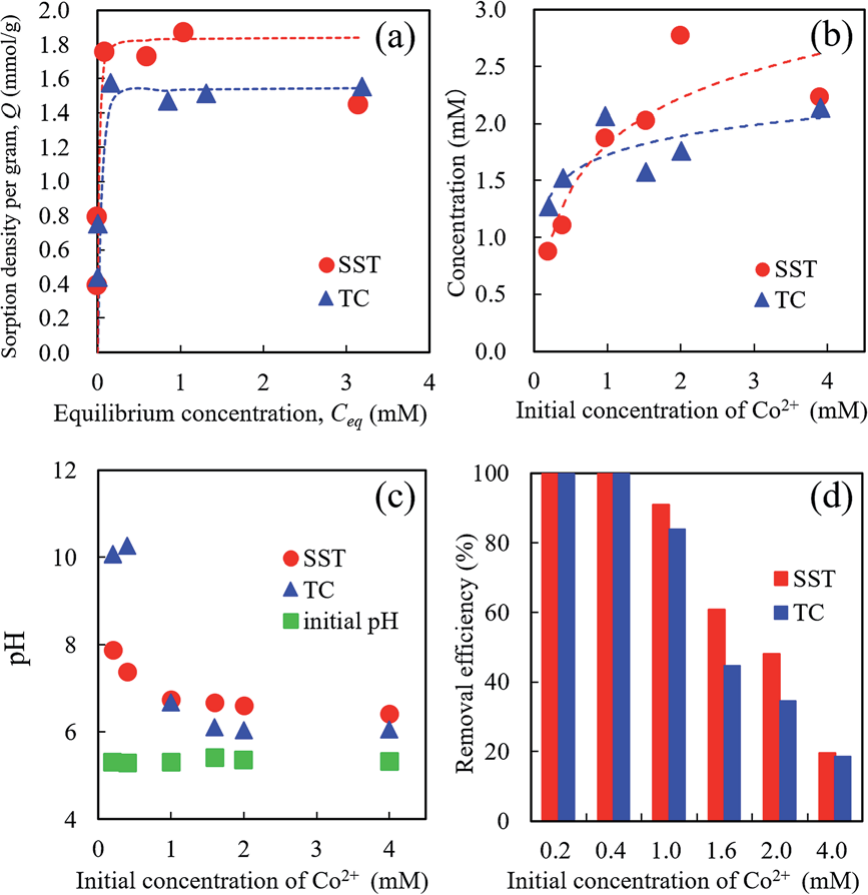
Co^2+^ Sorption behaviour of SST and TC. (a) Sorption isotherm of Co^2+^ on sorbents, (b) released Na^+^ concentration, (c) pH changes of the test solution, and (d) removal efficiency of Co^2+^ after sorption testing using 0.2–4.0 mM cobalt nitrate solution.

The Co^2+^ sorption test of SST was performed by batch testing using 0.2–4.0 mM aqueous solutions of cobalt nitrate (Fig. 7). Compared with TC, the SST sample showed a higher sorption density of Co^2+^ (Fig. 7a). The sorption isotherm of Co^2+^ was fitted to the Langmuir model, and the results of the theoretical analysis are shown in Table 1. The maximum sorption density (*Q*_max_) of SST was higher than that of TC. The sorption mechanism of the layered sodium titanate is known to be an ion-exchange reaction of cations with Na^+^ in the interlayers.^20–22^ Therefore, the change in the concentration of Na^+^ released in the solution after the sorption testing was also investigated (Fig. 7b). The concentrations of the released Na^+^ from SST and TC were approximately the same. As the initial concentration of Co^2+^ in the test solution increased, the number of released Na^+^ also increased. In addition, the concentration of the Na^+^ released from the sorbents was higher than the concentration of the incorporated Co^2+^ from the test solution.

**Table 1:** Langmuir isotherm constants for Co^2+^ adsorption on SST and TC

Sample	*Q*_max_ (mmol g^−1^)	*K* (L mmol^−1^)	*r* ^2^
SST	1.84	154.1	0.994^a^
TC	1.55	84.8	0.997

a SST was calculated excluding the data from the sorption testing with an initial concentration of 4.0 mM cobalt nitrate.

As shown in Fig. 7c, the pH value of the test solution before the sorption test was approximately 5.5, regardless of the Co^2+^ concentration. After the sorption test, the solution pH increased. The final pH values decreased as the initial concentration of Co^2+^ in the test solution increased. The pH values of SST and TC in the low concentration (0.2 and 0.4 mM cobalt nitrate) test solutions after the sorption test were 8 and 10, respectively. This increase in pH indicates that ion-exchange occurred between the H^+^ in the solution and Na^+^ in the SST or TC.

The removal efficiencies of SST and TC at 0.2 and 0.4 mM were 100% (Fig. 7d). These results mean that the number of released Na^+^ was higher than that of the Co^2+^ incorporated into the sorbents. At initial concentrations of 1.0–2.0 mM cobalt nitrate, the removal efficiency of SST was higher than that of TC. These results indicate that SST has a greater ability of Co^2+^ removal than TC.

To investigate the Co^2+^ sorption behaviour of the sorbents, XRD analysis, SEM observation, and elemental analysis by energy-dispersive X-ray (EDX) analysis of the residues were also performed. Fig. S7† shows the XRD patterns of the sorbents after sorption testing at 2.0 and 4.0 mM cobalt nitrate. The diffraction peaks of SST before (Fig. 1a) and after the sorption test (Fig. S7a†) were identical. In the case of the TC sample, the typical TC peaks were absent and the new peaks were identified as H_2_Ti_3_O_7_ (PDF no. 00-031-1329) after the sorption test (Fig. S7b†).

**Fig. 8:**
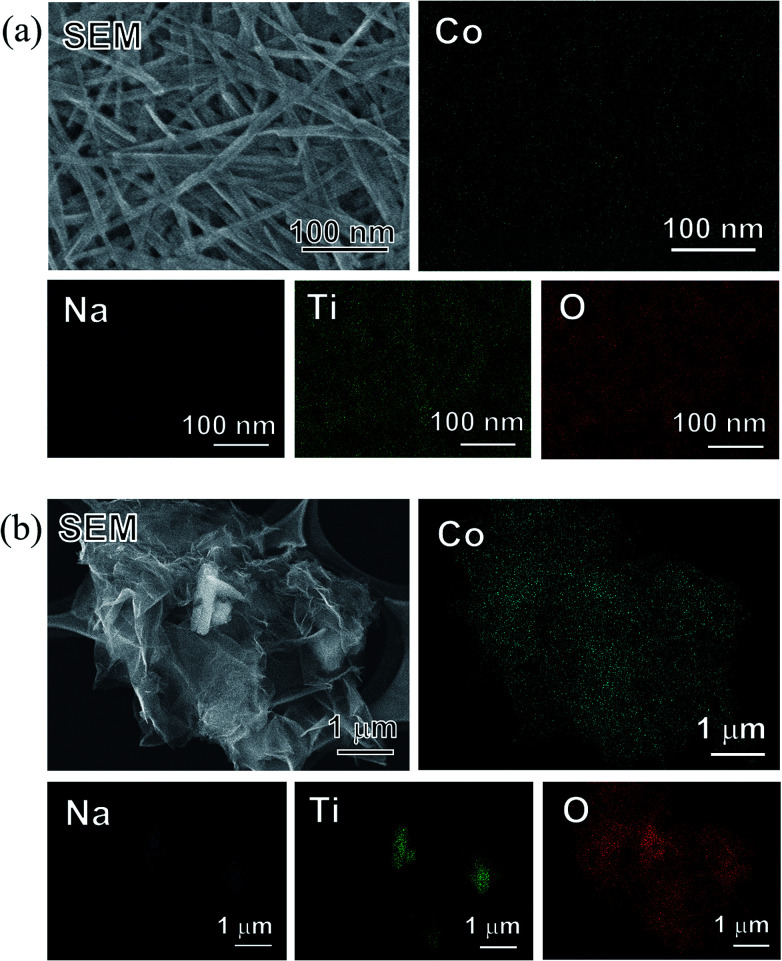
SEM images and EDX mappings of (a) SST and (b) TC sorbents after the Co^2+^ sorption test at 4.0 mM cobalt nitrate.

From the SEM images (Fig. 8a), no precipitation was observed on the surface of the SST samples. In addition, the unique mat structure, which looks like a non-woven fabric, remained after the sorption tests (Fig. 8a). In contrast, precipitates were observed on the TC particles, and the Co and O elemental images clearly overlapped with the amorphous precipitate (Fig. 8b). Ti and Na were detected in the TC particle site.

**Fig. 9:**
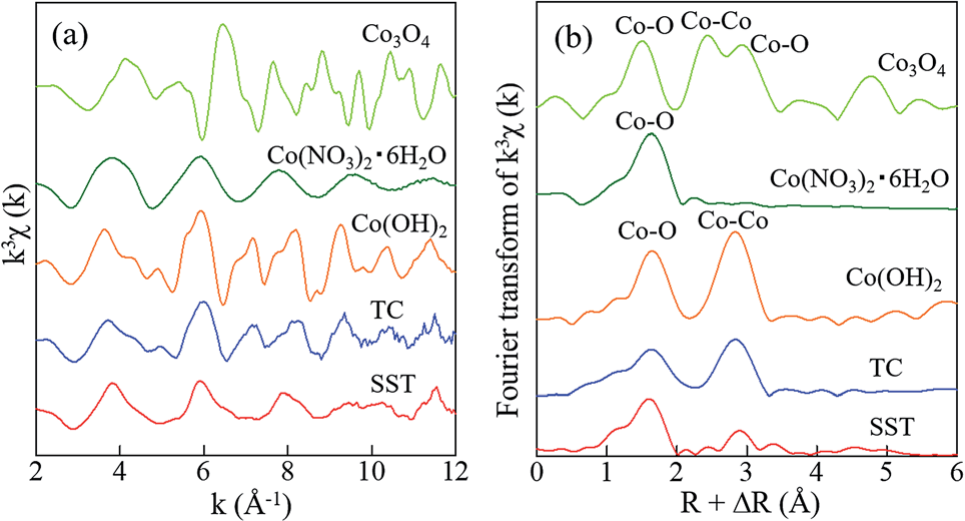
Spectra of (a) the Co K-edge *k*^3^-weighted EXAFS, and (b) FT-EXAFS of standard reagents and SST. The radial distribution functions were not corrected for phase shift.

In addition, the local environments of Co in the sorbents were investigated by EXAFS analysis. The Co K-edge EXAFS and FT-EXAFS spectra of SST and TC after sorption and the reference samples are presented in Fig. 9. The Co–O peak, as seen in the cobalt oxide reference sample, is the first coordination shell and is also detected in the SST and TC samples (Fig. 9b). The FT-EXAFS spectra of SST were similar to the cobalt nitrate reference sample. However, the FT-EXAFS spectra of TC were similar to the cobalt hydroxide reference sample. From these results, Co^2+^ was incorporated into the SST interlayer by the ion-exchange reaction, where it remained in an ionic state in the structure. In contrast, a cobalt hydroxide precipitate covered the TC particles after the sorption test, which resulted in a dramatic increase in the solution pH of the TC samples (Fig. 7c). Therefore, in the case of TC, Co^2+^ was both incorporated by ion-exchange and precipitated as cobalt hydroxide.

The Co^2+^ sorption test of the SST_*t* samples was also performed. A comparison of the initial Na/Ti molar ratio and the Co/Ti molar ratio after the test is presented in Fig. S8.† The Co/Ti molar ratio of the SST_*t* sample dramatically increased to 0.27 at a heating time of 1 h, and then the ratio decreased to 0.25 as the hydrothermal synthesis time increase to 12 h. Then, the Co/Ti molar ratio increased up to 0.29 as the heating time increased. This result indicates that the changes in the Co/Ti molar ratio can be categorised into three stages due to the difference in synthesis time. Taking the XRD results (Fig. 4) and initial Na/Ti molar ratio (Fig. S8†) into consideration, the observed increase in the Co/Ti molar ratio of SST_*t* after 1 h of heating time indicates the rise of the ion-exchange reaction due to the formation of a layered structure. This is because the peak intensity of the SST_1 sample clearly increased compared with that of the SST_0 sample (Fig. 4a). After that, the interlayer distance decreased as the heating time was increased to 12 h (Fig. 4b), and the Co/Ti molar ratio decreased slightly. During this time, the initial Na/Ti molar ratio increased (Fig. S8†). Therefore, although the concentration of Na in the structure increased, it can be seen that the incorporation of Co^2+^ into the structure was slightly suppressed due to the decrease in the distance between layers. Furthermore, it is considered that the Co/Ti molar ratio slightly increased after stabilising the crystallinity of SST by increasing the heating time above 12 h.

### Sorption mechanism of Co^2+^ on SST and TC

In this study, the Co^2+^ sorption behaviour of the SST mat that was synthesised *via* the template-free alkaline hydrothermal synthesis was investigated in comparison with TC. Previously, researchers studied the sorbents for Co^2+^ removal, such as sodium titanates, zeolites, hydroxyapatite, and clay minerals.^4–13^ In comparison with past reports, among the adsorbents composed of inorganic materials, SST has shown a relatively high sorption capacity for Co^2+^.

There are two possible reasons why the sorption capacity of SST was higher than TC. One reason is that the SSA of SST was higher than that of TC due to their respective crystal morphologies. As mentioned above, the SST mat consisted of nanofibres, which are expected to increase the number of ion-exchange sites due to the high SSA and low crystallinity. Therefore, although the zeta potential of SST is slightly higher than that of TC (Fig. S3†) at the pH value of the test solution, many cations might be incorporated in SST due to its unique nanostructure.

Another reason is that the elemental analysis and the crystal structure of SST determined by XRD confirm the presence of a dititanate phase in SST, which has a high sorption capacity and consists of lamellar structures.^17^ In contrast, the crystal structure of TC was determined to be a trititanate phase with a zigzag layer structure and a smaller interlayer distance than that of sodium dititanate. Researchers have previously proposed that the ease of ion-exchange varies with different Na sites.^32^ By comparing the concentration only, the concentration of Na (wt%) in SST is lower than that in TC (Table 2). Therefore, the theoretical IEC of SST and TC was calculated based on their Na concentration to be 3.62 and 5.62 mmol g^−1^, respectively. In the case of SST, IEC/2 and *Q*_max_ were equivalent (Table 1, Table 2). This result indicates that the Na^+^ contained in SST was almost ion-exchanged to Co^2+^ in the solution. In contrast, the *Q*_max_ of TC was half of the value of IEC/2 (Table 1, Table 2). That is because the ion-exchange occurred between the Na^+^ contained in TC with Co^2+^ and H^+^ in the solution. This is the reason why the final pH was as high as 10 at a low initial Co^2+^ concentration (0.2–0.4 mM). At a higher initial Co^2+^ concentration, cobalt hydroxide was precipitated around TC due to the local increase in pH around TC, and this inhibited the ion-exchange reaction. Moreover, sodium trititanate (like TC) does not easily undergo ion-exchange with Co^2+^. This is because the trititanate structure has Na^+^ that are difficult to exchange, as mentioned above.^32^ Therefore, SST had higher Co^2+^ sorption capacity than TC, although SST showed only the ion-exchange reaction. Thus, differences in the crystal structure of sodium titanate can affect the ion-exchange behaviour with Co^2+^.

**Table 2:** Na and Ti concentrations in SST and TC by ICP-OES analysis, and ion-exchange capacity (IEC) calculated by Na concentration of SST and TC

Sample	Na (wt%)	Ti (wt%)	IEC/2 (mmol g^−1^)
SST	8.34	38.8	1.81
TC	12.9	46.3	2.81

To enhance the ion-exchange property of the layered sodium titanate, in addition to the impact of the crystal structure and morphology, the Na concentration of titanate that undergoes ion-exchange with the target cation needs to be controlled. This new finding will contribute to the development of high-performance titanates that are based on ion exchangers in the future.

### Sorption selectivity of SST and TC

**Fig. 10:**
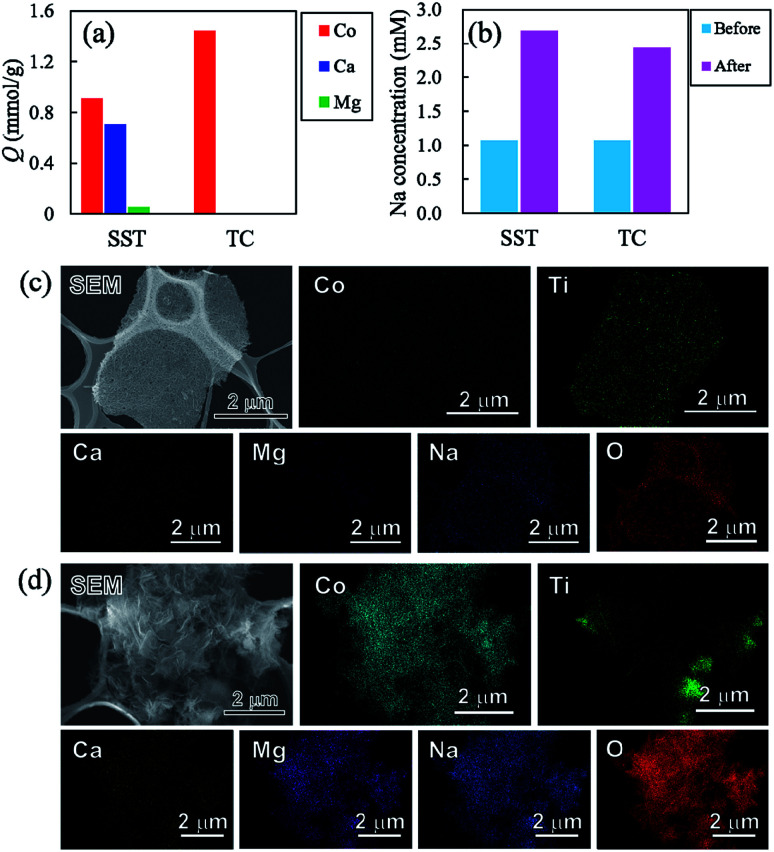
(a) Sorption density per gram (*Q*) of Co, Ca, and Mg on samples, (b) Na concentration from samples, and SEM images of (c) SST and (d) TC after the sorption test using the test solution of four-element coexistence.

To investigate the sorption selectivity of ions on SST and TC, the selectivity test of Co^2+^ was conducted in comparison with the sorption selectivities of Ca^2+^, Mg^2+^, and Na^+^. The amount of sorption of each element on SST and TC is presented in Fig. 10a. The sorption of SST was on the order of Co^2+^ > Ca^2+^ > Mg^2+^, while TC showed only Co^2+^ sorption. The amount of sorption of all elements on SST is approximately 1.67 mmol g^−1^, which is almost the same as the Co^2+^ sorption value tested at 1 mM, as shown in Fig. 7a. Therefore, Ca^2+^ and Mg^2+^ are assumed to be incorporated into the SST structure *via* ion-exchange. Fig. 10b shows the Na concentration of the solutions before and after the sorption test. An increase in the Na content was observed in both samples, which indicated that Na^+^ had been released from SST and TC. On the other hand, the solution pH values of SST and TC were 6.86 and 6.66, respectively. These pH values were higher than the initial test solution (pH = 5.35). Therefore, to investigate the formation of the precipitates on the samples, the SEM-EDX observation was performed after the sorption test. As shown in Fig. 10c and d, although there was no precipitate on SST, a precipitate was clearly observed on the TC surface. Based on the XRD analysis, the identity of the precipitate was inconclusive (Fig. S9†). However, it was most likely identified as cobalt hydroxide, as observed in the EDX maps, because the Co and O images overlapped with the location of the precipitate in the SEM image. The Ca and Mg images also overlapped with those of Co and O. The adsorption of Ca^2+^ and Mg^2+^ might have occurred in a small amount because the sorption of these elements on TC was not observed *via* ICP-OES analysis (Fig. 10a). Furthermore, Na was detected on TC, as observed in the case of the Co^2+^ sorption test. In addition, Cl^−^ was not detected on either sample. That is, TC showed higher Co^2+^ sorption than that of SST because of the precipitation of cobalt hydroxide on TC. This was driven by the increase in pH that resulted from the ion-exchange of Na^+^ with H^+^. The solubility product constants (*K*_sp_) of Co(OH)_2_, Ca(OH)_2_, and Mg(OH)_2_ are 1.3 × 10^−15^, 5.5 × 10^−6^, and 1.1 × 10^−11^, respectively. Hence, TC showed high selectivity of Co^2+^, depending on its ease of precipitation as the hydroxide in comparison with Ca^2+^ and Mg^2+^.

## Conclusions

In this study, an SST mat composed of nanofibres was successfully synthesised by a facile template-free alkaline hydrothermal process using titanium sulphate. The Co^2+^ sorption property of SST was examined using batch testing in comparison with TC. From the X-ray analysis and SEM observations, SST was confirmed to have a layered dititanate phase, and it was shown that the mat structure resembles a non-woven fabric composed of nanofibres. The Co^2+^ sorption capacity of SST was higher than that of TC, and the sorption isotherm was fitted with the Langmuir model. The sorption mechanism of SST was determined to be an ion-exchange reaction with Na^+^. In contrast, the TC sample showed both the ion-exchange reaction and precipitation of cobalt hydroxide, as shown in the SEM images and FT-EXAFS analysis, which was due to the high concentration of Na^+^ in the TC sample. In the case of TC, the pH of the solutions increased rapidly due to the ion-exchange reaction with Na^+^ and H^+^. Based on the selectivity test, both samples showed high Co^2+^ sorption in the presence of Na^+^, Ca^2+^ and Mg^2+^. However, in the case of SST, this was due to an ion-exchange reaction, while for TC it was based on precipitation. These results indicate that the control of the crystal structure and morphology of a sorbent that undergoes ion-exchange with target cations is an important factor in the material design to achieve high permeability and high sorption properties.

## Conflicts of interest

There are no conflicts to declare.

## Supplementary Material

RA-010-D0RA06662A-s001

## References

[cit1] Faroon O. M. , Abadin H., Keith S., Osier M., Chappell L. L., Diamond G. and Sage G., Toxicological profile for cobalt, 2004

[cit2] Ahmadpour A., Tahmasbi M., Bastami T. R., Besharati J. A. (2009). Rapid removal of cobalt ion from aqueous solutions by almond green hull. J. Hazard. Mater..

[cit3] Dambies L., Jaworska A., Zakrzewska-Trznadel G., Sartowska B. (2010). Comparison of acidic polymers for the removal of cobalt from water solutions by polymer assisted ultrafiltration. J. Hazard. Mater..

[cit4] Rodríguez A., Sáez P., Díez E., Gómez J. M., García J., Bernabé I. (2018). Highly efficient low-cost zeolite for cobalt removal from aqueous solutions: characterization and performance. Environ. Prog. Sustainable Energy.

[cit5] Luo W., Bai Z., Zhu Y. (2018). Fast removal of Co(II) from aqueous solution using porous carboxymethyl chitosan beads and its adsorption mechanism. RSC Adv..

[cit6] Roosendael S. V., Onghena B., Roosen J., Michielsen B., Wyns K., Mullens S., Binnemans K. (2019). Recovery of cobalt from dilute aqueous solutions using activated carbon–alginate composite spheres impregnated with Cyanex 272. RSC Adv..

[cit7] Yavuz Ö., Altunlaynak Y., Güzel F. (2003). Removal of copper, nickel, cobalt and manganese from aqueous solution by kaolinite. Water Res..

[cit8] Qiu W., Zheng Y. (2009). Removal of lead, copper, nickel, cobalt, and zinc from water by a cancrinite-type zeolite synthesized from fly ash. Chem. Eng. J. (Amsterdam, Neth.).

[cit9] Smičiklas I., Dimović S., Plećaš I., Mitrić M. (2006). Removal of Co^2+^ from aqueous solutions by hydroxyapatite. Water Res..

[cit10] Rengaraj S., Yeon K. H., Kang S. Y., Lee J. U., Kim K. W., Moon S. H. (2002). Studies on adsorptive removal of Co(II), Cr(III) and Ni(II) by IRN77 cation-exchange resin. J. Hazard. Mater..

[cit11] Li D. M., Li F. Z., Liao J. L., Yang J. J., Li B., Chen Y. M., Yang Y. Y., Zhang J. S., Tang J., Liu N. (2016). Efficient removal of Co(II) from aqueous solution by titanate sodium nanotubes. Nucl. Sci. Tech..

[cit12] Nunes L. M., Cardoso V. D. A., Airoldi C. (2006). Layered titanates in alkaline, acidic and intercalated with 1,8-octyldiamine forms as ion-exchangers with divalent cobalt, nickel and copper cations. Mater. Res. Bull..

[cit13] Cardoso V. D. A., de Souza A. G., Sartoratto P. P., Nunes L. M. (2004). The ionic exchange process of cobalt, nickel and copper(II) in alkaline and acid-layered titanates. Colloids Surf., A.

[cit14] Veselý V., Pekárek V. (1972). Synthetic inorganic ion-exchangers—I: hydrous oxides and acidic salts of multivalent metals. Talanta.

[cit15] Lehto J., Koivula R., Leinonen H., Tusa E., Harjula R. (2019). Removal of Radionuclides from Fukushima Daiichi Waste Effluents. Sep. Purif. Rev..

[cit16] Takahatake Y., Shibata A., Nomura K., Sato T. (2014). Effect of flowing water on Sr sorption changes of hydrous sodium titanate. Minerals.

[cit17] Zhao B., Lin L., He D. (2013). Phase and morphological transitions of titania/titanate nanostructures from an acid to an alkali hydrothermal environment. J. Mater. Chem. A.

[cit18] Sun X., Chen X., Li Y. (2002). Large-scale synthesis of sodium and potassium titanate nanobelts. Inorg. Chem..

[cit19] Štengl V., Bakardjieva S., Šubrt J., Večerníková E., Szatmary L., Klementová M., Balek V. (2006). Sodium titanate nanorods: preparation, microstructure characterization and photocatalytic activity. Appl. Catal., B.

[cit20] Yang D., Sarina S., Zhu H., Liu H., Zheng Z., Xie M., Smith S. V., Komarneni S. (2011). Capture of radioactive cesium and iodide ions from water by using titanate nanofibers and nanotubes. Angew. Chem., Int. Ed..

[cit21] Filipowicz B., Pruszyński M., Krajewski S., Bilewicz A. (2014). Adsorption of ^137^Cs on titanate nanostructures. J. Radioanal. Nucl. Chem..

[cit22] Goto T., Cho S. H., Lee S. W., Sekino T. (2018). Sorption capacity of Cs^+^ on titania nanotubes synthesized by solution processing. J. Ceram. Soc. Jpn..

[cit23] Meng X. D., Wang D. Z., Liu J. H., Zhang S. Y. (2004). Preparation and characterization of sodium titanate nanowires from brookite nanocrystallites. Mater. Res. Bull..

[cit24] Suzuki Y., Yoshikawa S. (2004). Synthesis and thermal analyses of TiO_2_-derived nanotubes prepared by the hydrothermal method. J. Mater. Res..

[cit25] Kasuga T., Hiramatsu M., Hoson A., Sekino T., Niihara K. (1998). Formation of titanium oxide nanotube. Langmuir.

[cit26] Matsuo S., Sakaguchi N., Wakita H. (2005). Pre-edge features of Ti K-edge X-ray absorption near-edge structure for the local structure of sol-gel titanium oxides. Anal. Sci..

[cit27] Wu Z. Y., Ouvrard G., Gressier P., Natoli C. R. (1997). Ti and O K edges for titanium oxides by multiple scattering calculations: comparison to XAS and EELS spectra. Phys. Rev. B: Condens. Matter Mater. Phys..

[cit28] Ma R., Fukuda K., Sasaki T., Osada M., Bando Y. (2005). Structural features of titanate nanotubes/nanobelts revealed by Raman, X-ray adsorption fine structure and electron diffraction characterizations. J. Phys. Chem. B.

[cit29] Farges F., Brown G. E. (1997). Ti-Edge XANES Studies of Ti Coordination and Disorder in Oxide Compounds: Comparison between Theory and Experiment. Phys. Rev. B: Condens. Matter Mater. Phys..

[cit30] Zhang Y., Jiang Z., Huang J., Lim L. Y., Li W., Deng J., Gong D., Tang Y., Lai Y., Chen Z. (2015). Titanate and Titania Nanostructured Materials for Environmental and Energy Applications: A Review. RSC Adv..

[cit31] Zhang D. R., Kim C. W., Kang Y. S. (2010). A Study on the Crystalline Structure of Sodium Titanate Nanobelts Prepared by the Hydrothermal Method. J. Phys. Chem. C.

[cit32] Yang D., Zheng Z., Liu H., Zhu H., Ke X., Xu Y., Wu D., Sun Y. (2008). Layered titanate nanofibers as efficient adsorbents for removal of toxic radioactive and heavy metal ions from water. J. Phys. Chem. C.

