# Supplementary material for: Sorption capacity of seaweed-like sodium titanate mats for Co2+ removal
Source: RSC Adv. 2020 Nov 11;10(67):41032–40. doi: 10.1039/d0ra06662a (PMC9057711; doi:10.1039/d0ra06662a)
Supplement: RA-010-D0RA06662A-s001 [file RA-010-D0RA06662A-s001.pdf]

Electronic Supplementary Material (ESI) for RSC Advances.
This journal is © The Royal Society of Chemistry 2020

Electronic Supplementary Information

Sorption capacity of seaweed-like sodium titanate mats for Co^{2+} removal

Yoshifumi Kondo,[ab] Tomoyo Goto,*[a] and Tohru Sekino*[a]

[a] The Institute of Scientific and Industrial Research (ISIR), Osaka University, 8-1 Mihogaoka, Ibaraki, Osaka 567-0047, Japan

[b] Division of Materials and Manufacturing Science, Graduate School of Engineering, Osaka University, 2-1 Yamadaoka, Suita, Osaka 565-0871, Japan

Table of contents

	Pages
**Fig. S1 – S9**	**S3 – S11**
**Table S1**	**S12**

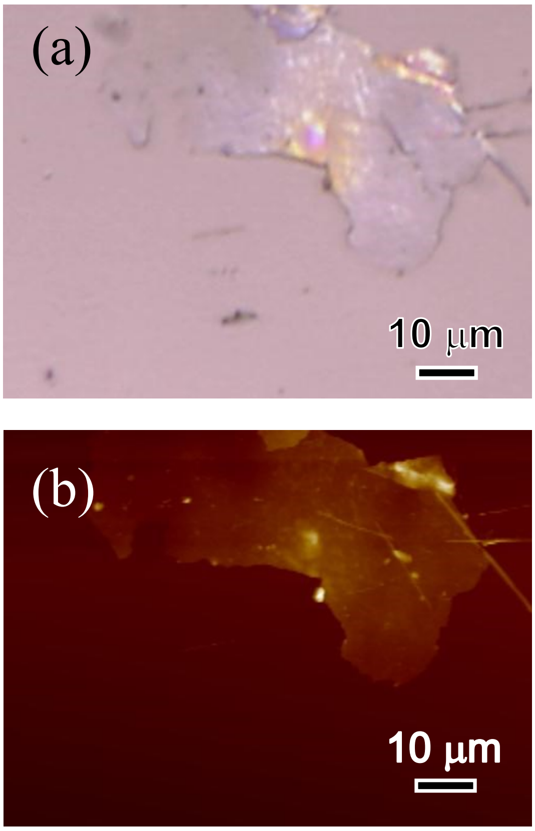

Fig. S1. (a) Optical microscope image and (b) AFM image of SST sample.

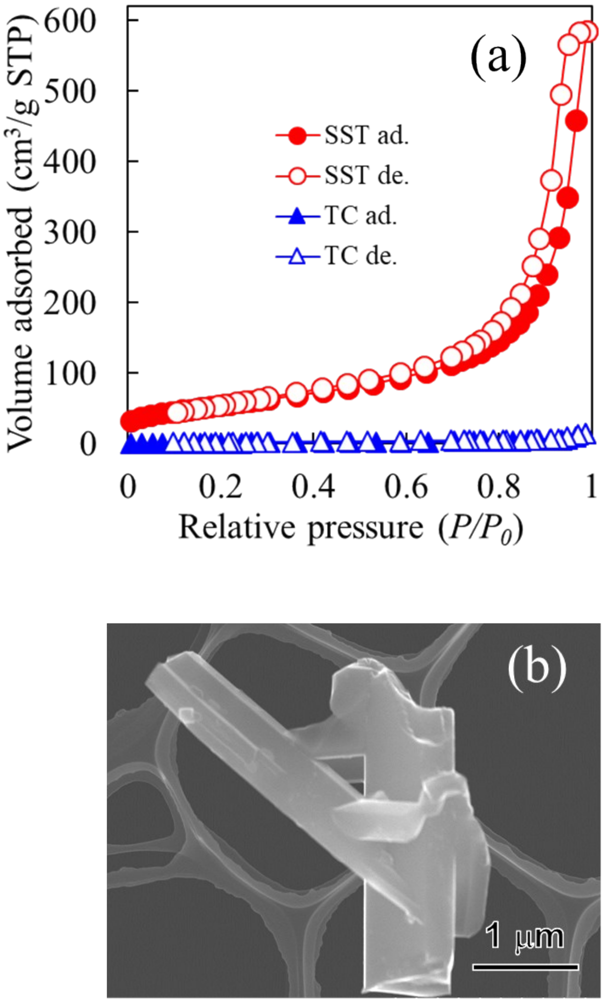

Fig. S2. (a) N_2 adsorption-desorption isotherms of SST and TC samples and (b) SEM image of TC sample.

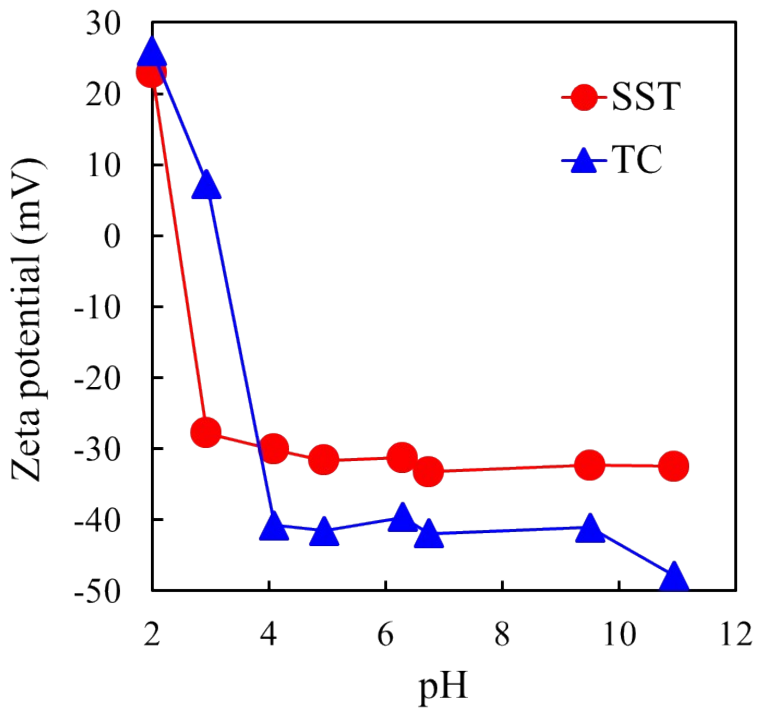

Fig. S3. Zeta potential against the solution pH of SST and TC samples at 25 °C.

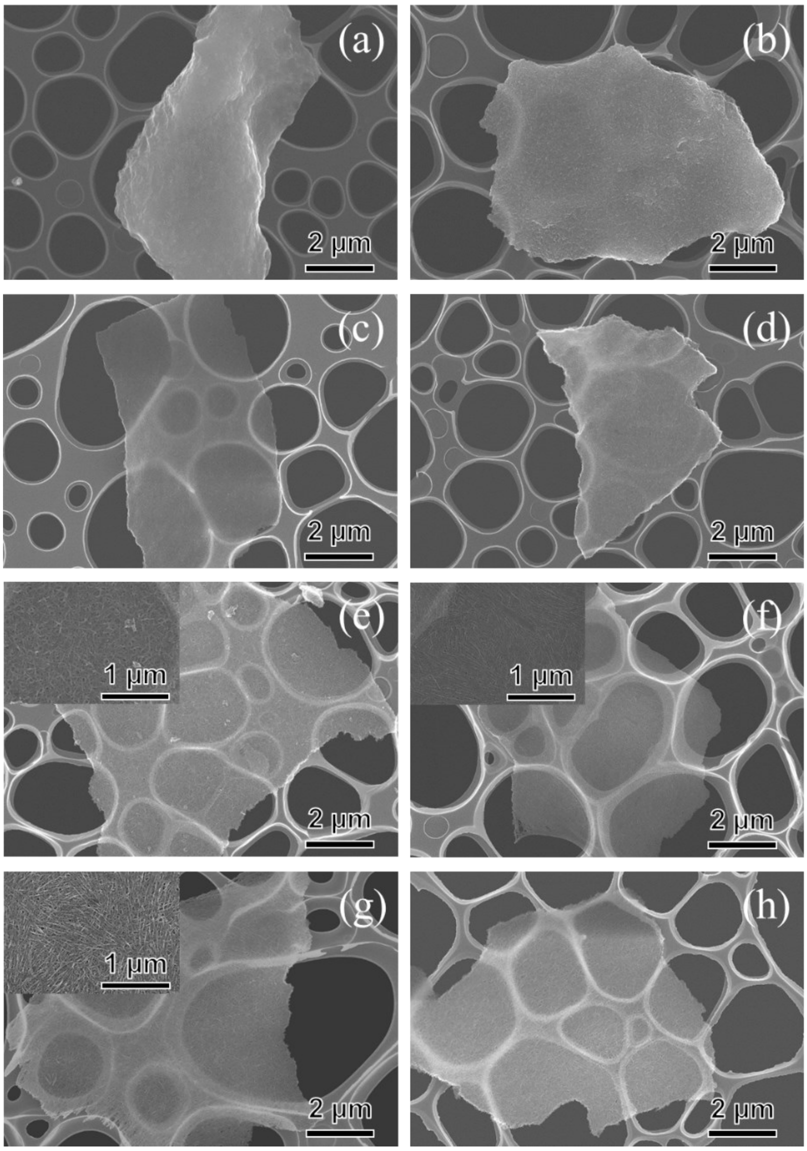

Fig. S4. SEM images of (a) SST_0, (b) SST_1, (c) SST_2, (d) SST_3, (e) SST_6, (f) SST_12, (g) SST_24, and (h) SST samples.

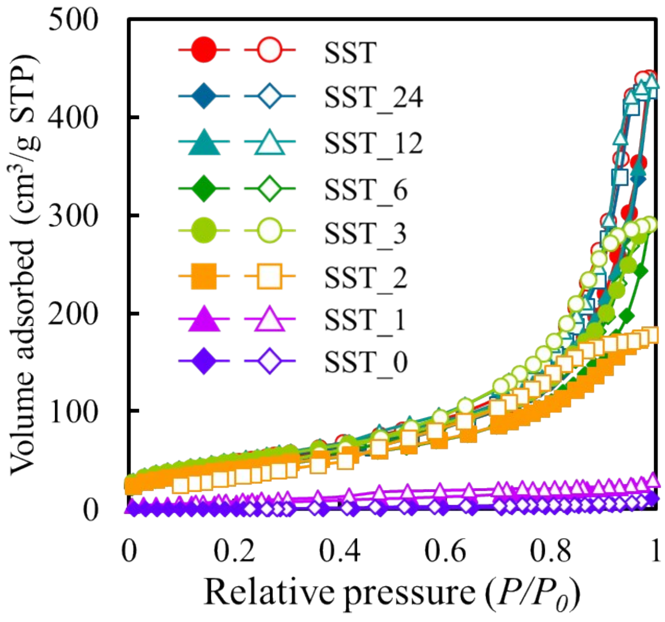

Fig. S5. N_2 adsorption-desorption isotherms of SST_X samples.

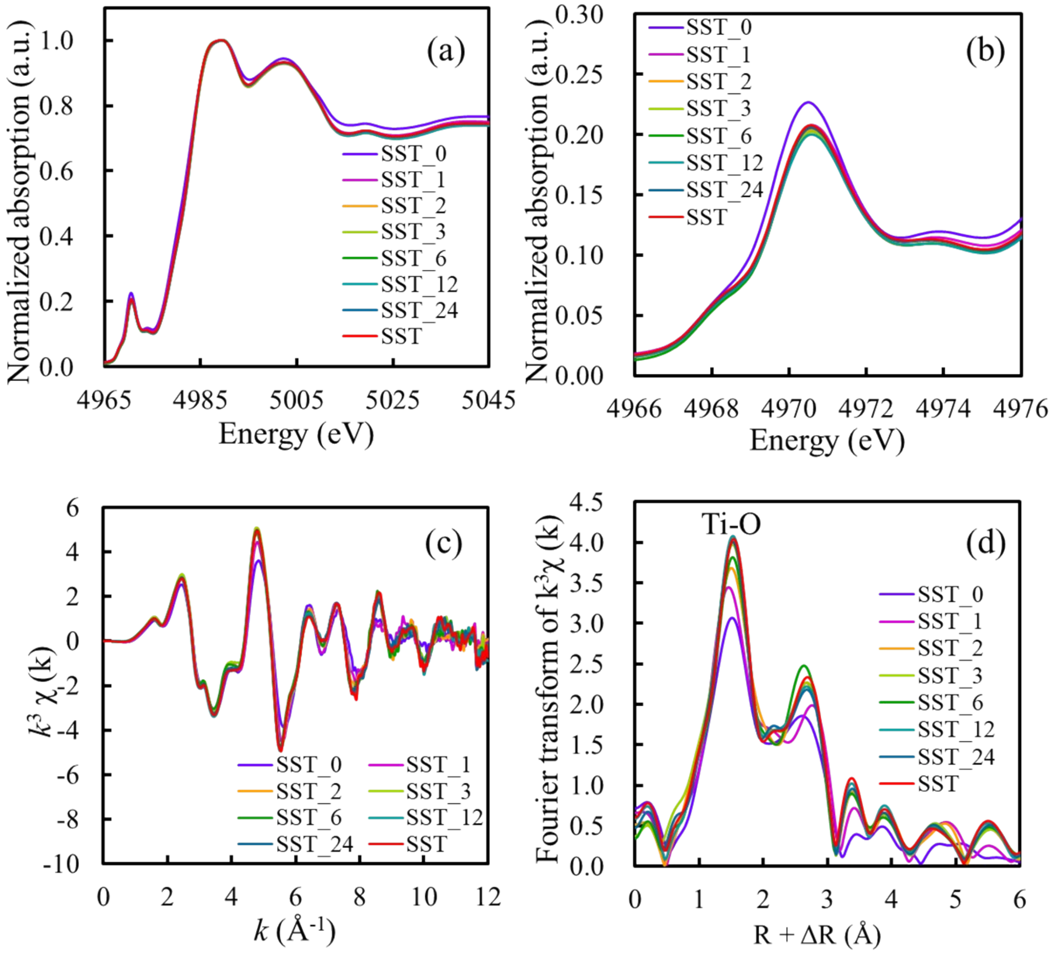

Fig. S6. Ti K-edge (a) XANES spectra, (b) pre-edge peaks, (c) EXAFS spectra, and (d) FT-EXAFS spectra of SST_X samples. Radial distribution functions were not corrected for phase shift.

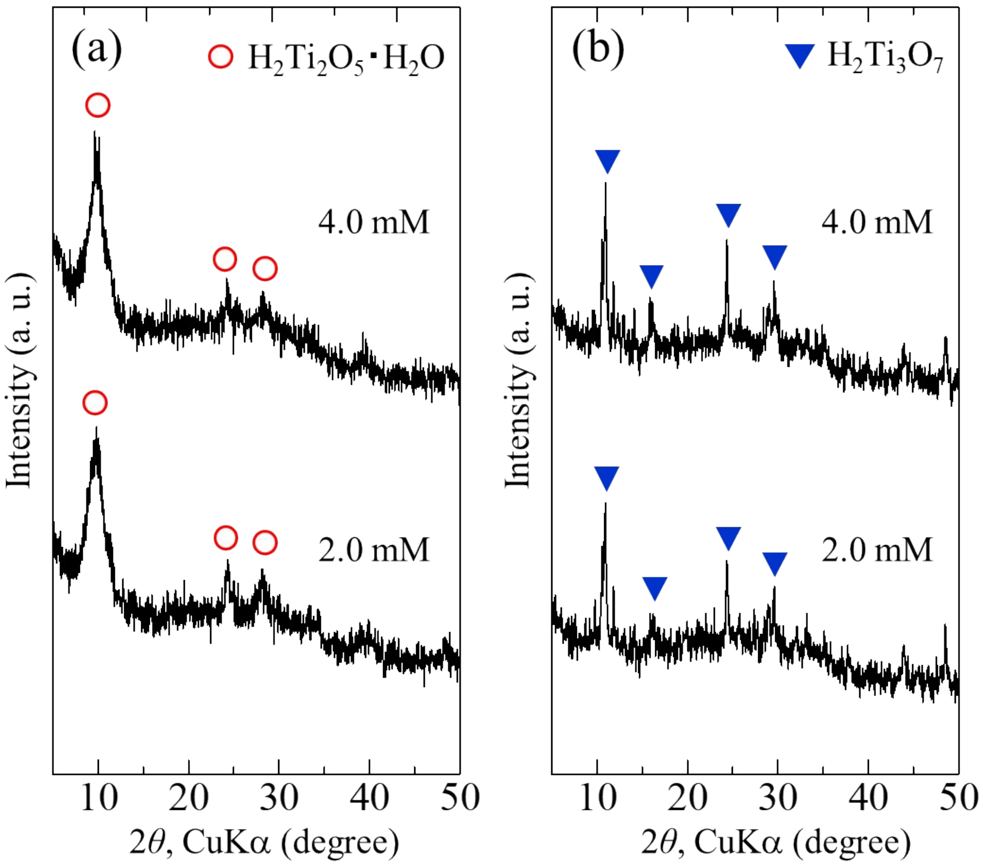

Fig. S7. XRD patterns of (a) SST and (b) TC samples after Co^{2+} sorption testing 2.0 mM and 4.0 mM cobalt nitrate.

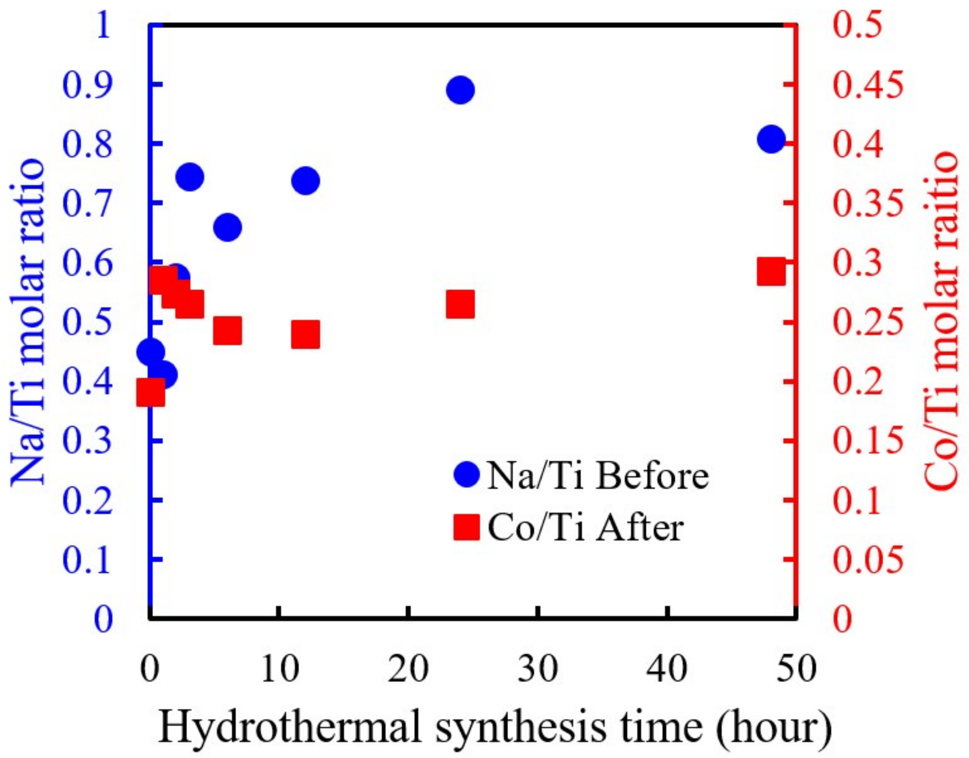

Fig. S8. Changes in Na/Ti molar ratio of SST_X samples before Co^{2+} sorption tests and Co/Ti molar ratio of SST_X samples after the tests.

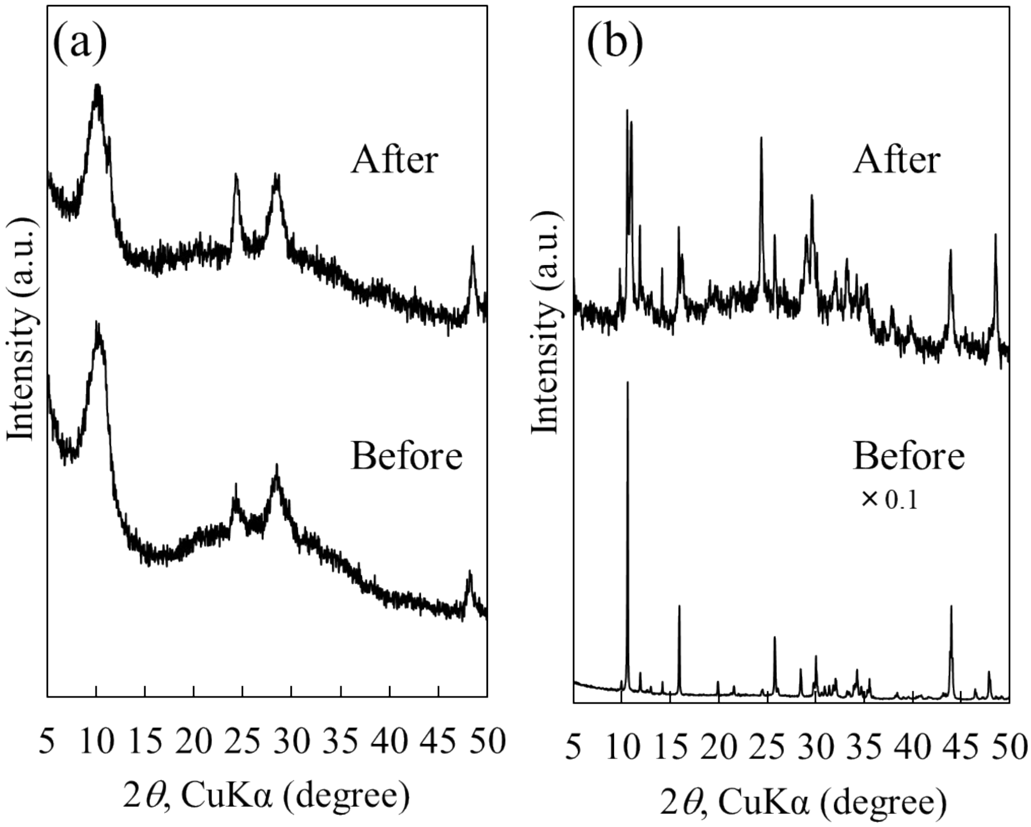

Fig. S9. XRD patterns of (a) SST and (b) TC samples after the sorption test of four-element coexistence (Co^{2+}, Ca^{2+}, Mg^{2+}, and Na^{+}).

Table S1. BET specific surface area of SST_X samples.

Sample	BET specific surface area [m^2/g]
SST_0	2.222
SST_1	25.23
SST_2	148.3
SST_3	178.8
SST_6	146.3
SST_12	184.2
SST_24	173.4
SST	158.3
